# Upregulation of ANGPTL6 in mouse keratinocytes enhances susceptibility to psoriasis

**DOI:** 10.1038/srep34690

**Published:** 2016-10-04

**Authors:** Hiroki Tanigawa, Keishi Miyata, Zhe Tian, Jun Aoi, Tsuyoshi Kadomatsu, Satoshi Fukushima, Aki Ogata, Naoki Takeda, Jiabin Zhao, Shunshun Zhu, Kazutoyo Terada, Motoyoshi Endo, Jun Morinaga, Taichi Sugizaki, Michio Sato, Masaki Suimye Morioka, Ichiro Manabe, Youichi Mashimo, Akira Hata, Yoshitaka Taketomi, Kei Yamamoto, Makoto Murakami, Kimi Araki, Masatoshi Jinnin, Hironobu Ihn, Yuichi Oike

**Affiliations:** 1 Department of Molecular Genetics, Graduate School of Medical Sciences, Kumamoto University, 1-1-1 Honjo, Chuo-ku, Kumamoto 860-8556, Japan; 2 Department of Dermatology and Plastic Surgery, Graduate School of Medical Sciences, Kumamoto University, 1-1-1 Honjo, Chuo-ku, Kumamoto 860-8556, Japan; 3 Department of Immunology, Allergy and Vascular Medicine, Graduate School of Medical Sciences, Kumamoto University, 1-1-1 Honjo, Chuo-ku, Kumamoto 860-8556, Japan; 4 Division of Developmental Genetics, Center for Animal Resources and Development, Kumamoto University, 2-2-1 Honjo, Chuo-ku, Kumamoto 860-0811, Japan; 5 Department of Cardiovascular Medicine, Graduate School of Medicine, University of Tokyo, 7-3-1 Hongo, Bunkyo-ku, Tokyo 113-8655, Japan; 6 Department of Public Health, Chiba University, 1-8-1 Inohara, Chuo-ku, Chiba 260-8670, Japan; 7 Lipid Metabolism Project, Tokyo Metropolitan Institute of Medical Science, 2-1-6 Kamikitazawa, Setagaya-ku, Tokyo 156-8506, Japan; 8 Faculty of Bioscience and Bioindustry, Tokushima University, 2-1 Minami-Josanjima, Tokushima 770-8506, Japan

## Abstract

Psoriasis is a chronic inflammatory skin disease marked by aberrant tissue repair. Mutant mice modeling psoriasis skin characteristics have provided useful information relevant to molecular mechanisms and could serve to evaluate therapeutic strategies. Here, we found that epidermal ANGPTL6 expression was markedly induced during tissue repair in mice. Analysis of mice overexpressing ANGPTL6 in keratinocytes (*K14-Angptl6* Tg mice) revealed that epidermal ANGPTL6 activity promotes aberrant epidermal barrier function due to hyperproliferation of prematurely differentiated keratinocytes. Moreover, skin tissues of *K14-Angptl6* Tg mice showed aberrantly activated skin tissue inflammation seen in psoriasis. Levels of the proteins S100A9, recently proposed as therapeutic targets for psoriasis, also increased in skin tissue of *K14-Angptl6* Tg mice, but psoriasis-like inflammatory phenotypes in those mice were not rescued by S100A9 deletion. This finding suggests that decreasing S100A9 levels may not ameliorate all cases of psoriasis and that diverse mechanisms underlie the condition. Finally, we observed enhanced levels of epidermal ANGPTL6 in tissue specimens from some psoriasis patients. We conclude that the *K14-Angptl6* Tg mouse is useful to investigate psoriasis pathogenesis and for preclinical testing of new therapeutics. Our study also suggests that ANGPTL6 activation in keratinocytes enhances psoriasis susceptibility.

Psoriasis is a chronic inflammatory skin disease marked by thickened epidermis and caused by hyper-proliferation of prematurely differentiated keratinocytes^1^,^2^,^3^. Psoriasis is a complex disease marked by several inflammatory phenotypes^1^,^2^. Symptomatic treatment to suppress epidermal proliferation and skin tissue inflammation has been available for several years^3^,^4^. However, since quality of life psoriasis patients declines due to changes their appearance and clinical symptoms of pain and itching^5^, the development of more effective therapeutics is necessary. Although specific molecular mechanisms underlying psoriasis remain unclear, its pathophysiology is recognized overall as involving an aberrant immune response in skin tissue accompanied by activated production of inflammatory cytokines^1^,^6^. Moreover, psoriasis increases the risk of developing other inflammatory diseases, such as psoriatic arthritis, Crohn’s disease, cardiovascular disease, and lymphoma^7^. Recently, immune-suppressive biological agents were developed as therapeutics for psoriasis. As anticipated, these agents are more effective than conventional therapies^3^,^8^ but can induce side effects associated with immune suppression, restricting their application^8^. Therefore, identification of factors driving psoriasis pathogenesis remains an urgent problem.

Efforts have been made to develop mouse models of human psoriasis in order to define underlying mechanisms and identify new drug targets^9^. For example, in mice, epidermal deletion of *JunB*, which in humans is localized to the so-called psoriasis susceptibility region *PSORS6,* along with deletion of its functional homologue *c-Jun,* leads to skin inflammation with histological and molecular hallmarks of human psoriasis. Skin tissues of *JunB/c-Jun* double-mutant (DKO) mice show elevated levels of S100A9 proteins, and in humans genes encoding these proteins are localized in the psoriasis susceptibility region *PSORS4*^10^. Moreover, psoriasis-like skin proliferative and inflammatory phenotypes are significantly rescued in DKO mice in which *S100A9* is also genetically deleted^11^, suggesting that S100A9 could be a useful therapeutic target for psoriasis.

Angiopoietin-like proteins (ANGPTLs) are proteins structurally similar to angiopoietin and marked by an N-terminal coiled-coil domain and a C-terminal fibrinogen-like domain^12^. Among them, ANGPTL6, also known as Angiopoietin-related growth factor (AGF), functions in proliferation of epidermal keratinocytes and in remodeling, repair and regeneration of skin tissue in mice^13^. The human ANGPTL6 gene is located at 19p13.2 in a region known as the psoriasis susceptibility region *PSORS6*^14^,^15^. These findings suggest a potential role for ANGPTL6 in psoriasis susceptibility, although this associated has not been documented.

In this study, we found that ANGPTL6 overexpression in keratinocytes of transgenic (*K14-Angptl6* Tg) mice promotes thickened epidermis marked by hyper-proliferation of prematurely differentiated keratinocytes and increased chemokine/cytokine expression, accelerating recruitment of neutrophils and endothelial cells to epidermis and contributing to phenotypic changes associated with psoriasis. Moreover, levels of S100A9 proteins also increased in skin tissue of *K14-Angptl6* Tg mice. Psoriasis-like skin phenotypes exhibited by mice mutant in S100A9 were not rescued on a *K14-Angptl6* Tg background. We also observed increased epidermal ANGPTL6 production in some psoriasis patients. To the best of our knowledge, this is the first report showing that increased ANGPTL6 activity in keratinocytes may enhance psoriasis susceptibility.

## Results

### *K14-Angptl6* Tg mice develop psoriasis-like epidermal proliferative and inflammatory phenotypes

**Figure 1:**
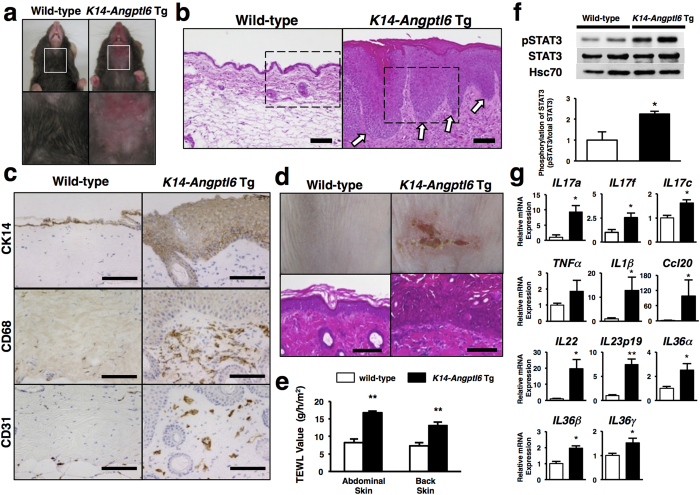
*K14-Angptl6* Tg mice exhibit a psoriasiform phenotype. (**a**) *K14-Angptl6* Tg mice (right panels), unlike comparably aged wild-type mice (left panels), spontaneously develop psoriasiform skin by 20 weeks of age. Squares in upper panels are magnified in corresponding lower panels. (**b**) HE-stained neck skin of wild-type (left) and *K14-Angptl6* Tg (right) mice at 20 weeks of age. Arrows indicate epidermal thickening with elongated rete ridges. Dotted boxes are magnified in Supplementary Fig. S1b. Scale bar: 100 μm. (**c**) CK14 (early keratinocyte marker), CD68 (macrophage marker) and CD31 (endothelial cell marker) staining of neck skin of wild-type (left) and *K14-Angptl6* Tg (right) mice at 20 weeks of age. Images are magnified from black-framed corresponding images in Supplementary Fig. S1c. Scale bar is 100 μm. (**d**) Psoriatic lesions of wild-type and *K14-Angptl6* Tg mice at day 4 after tape stripping injury (upper). Lower panels show HE-stained sections of skin tissues from these mice. Images are magnified from black-framed corresponding images shown in Supplementary Fig. S1d. Scale bar: 100 μm. (**e**) Transepidermal water loss (TEWL) values in indicated skin tissues from wild-type and *K14-Angptl6* Tg mice at 12 weeks of age (n = 6 per each group). (**f**) Representative immunoblotting and quantitation of ratio of p-STAT3 to total STAT3 from skin samples of wild-type and *K14-Angptl6* Tg mice at 20 weeks of age. Hsc70 served as loading control. Values in wild-type mice were set to 1 (n = 4 per each group). (**g**) Quantitative real-time RT-PCR analysis of indicated cytokine/chemokine transcripts in ear tissues at 20 weeks of age. Values for respective WT mice were set to 1 (n = 4–5 per each group). Data are means ± SEM. *p < 0.05, **p < 0.01 between genotypes or groups.

To assess a potential relationship between ANGPTL6 expression in skin tissue and psoriasis-like conditions, we evaluated phenotypes in *K14-Angptl6* Tg mice. We observed no gross difference in skin appearance between *K14-Angptl6* Tg and wild-type littermates from birth to 8 weeks of age. However, by 12 weeks skin tissue of *K14-Angptl6* Tg mice was reddish and swollen (Fig. 1a and Supplementary Fig. S1a). Histological analysis revealed a significantly thickened epidermal layer with elongated rete ridges, or thickenings, extending between dermal papillae (Fig. 1b and Supplementary Fig. S1b), and by 20 weeks *K14-Angptl6* Tg mice exhibited skin papillomatosis. Immunohistochemical analysis revealed cells positive for CK14, a marker of proliferating keratinocytes, in suprabasal and basal layers of skin tissue from *K14-Angptl6* Tg mice (Fig. 1c and Supplementary Fig. S1c), suggesting that ANGPTL6 overexpression in keratinocytes promotes proliferation of prematurely differentiated keratinocytes. Moreover, numerous CD68-positive macrophages and CD31-positive endothelial cells were recruited to the dermal layer of *K14-Angptl6* Tg skin tissue (Fig. 1c and Supplementary Fig. S1c), changes not seen in wild-type littermates. Interestingly, it is reported that skin tissues of *K14-Angptl6* mice show proliferation of CD31-positive endothelial cells and that Angptl6 enhances blood flow by promoting angiogenesis and arteriogenesis^16^,^17^.

Appearance of a rash or lesion after application of physical stimulus to healthy skin tissue, known as the Koebner phenomenon, is a common diagnostic for psoriasis^18^. We evaluated skin of *K14-Angptl6* Tg mice at 20 weeks of age for a potential Koebner effect in response to tape stripping. Following this stimulus, *K14-Angptl6* Tg mice developed a rash with skin exfoliation by 4 day after the procedure, changes not seen in similarly tested wild-type littermate control mice (Supplementary Fig. S1d). Histological examination of rash lesions revealed keratinocyte hyperplasia and neutrophil accumulation in the dermal layer (Fig. 1d and Supplementary Fig. S1d), physiological responses associated with human psoriasis.

Epidermal water barrier function is also disrupted in the active phase of psoriasis^19^,^20^; thus we examined related phenotypes in skin tissue of *K14-Angptl6* Tg mice by estimating transepidermal water loss (TEWL). TEWL values significantly increased in *K14-Angptl6* Tg mice relative to wild-type littermates, indicative of decreased epidermal water barrier function in transgenics (Fig. 1e). Moreover, STAT3 is reportedly activated in skin cells of psoriasis patients^21^, and transgenic mice expressing constitutively active STAT3 in keratinocytes represent a mouse model of psoriasis^21^. Our western blotting analysis revealed that the ratio of phosphorylated to total STAT3 protein increased in skin tissue of *K14-Angptl6* Tg relative to wild-type littermates (Fig. 1f), suggesting STAT3 activation in *K14-Angptl6* Tg mice. STAT3 signaling reportedly increases expression of IL17a, IL17f and Ccl20, chemokines/cytokines upregulated in psoriasis skin tissue^22^. RT-PCR analysis revealed that levels of transcripts encoding all three factors significantly increased in skin tissue of *K14-Angptl6* Tg relative to wild-type littermates (Fig. 1g and Supplementary Fig. S1e). We conclude overall that *K14-Angptl6* Tg mice exhibit psoriasis-like epidermal characteristics.

### ANGPTL6 expression is induced by physical injury to skin

Aberrant skin tissue repair is part of the pathogenesis of psoriasis^3^, and psoriasis can be exacerbated by physical stimuli such as sunburn, physical injury, or chemical irritants. Therefore, we asked whether ANGPTL6 expression levels in skin change in response to injury caused by repeated application and removal of adhesive tape in wild-type mice at 12 weeks of age. Levels of ANGPTL6 transcripts significantly increased at the injury site relative to uninjured control mice by 3 day after injury (Supplementary Fig. S1f). Also, ANGPTL6 protein levels significantly increased at day 1 after injury and remained higher that controls but to a lesser extent on day 3 (Supplementary Fig. S1g), suggesting that physical injury to skin induces ANGPTL6 expression. To identify cell types expressing ANGPTL6, we performed double-immunofluorescence analysis of skin tissues for ANGPTL6 and the macrophage marker Mac-3 following injury. We observed numerous macrophages infiltrating the dermis by 1 day after injury, a period corresponding to the greatest increase in ANGPTL6 protein level, and most infiltrated macrophages were ANGPTL6-positive (Supplementary Fig. S1h). Some neutrophils and T and B cells were detected in injured skin, and they also were ANGPTL6-positive (Supplementary Fig. S1i). Keratinocytes observed in these preparations were not ANGPTL6-positive (data not shown). These data indicate that in normal mouse skin, infiltrated macrophages, neutrophils, and T and B cells in dermis produce ANGPTL6 in response to injury.

### Increased ANGPTL6 activity in keratinocytes promotes psoriasis-like skin phenotypes independent of S100A9 function

**Figure 2:**
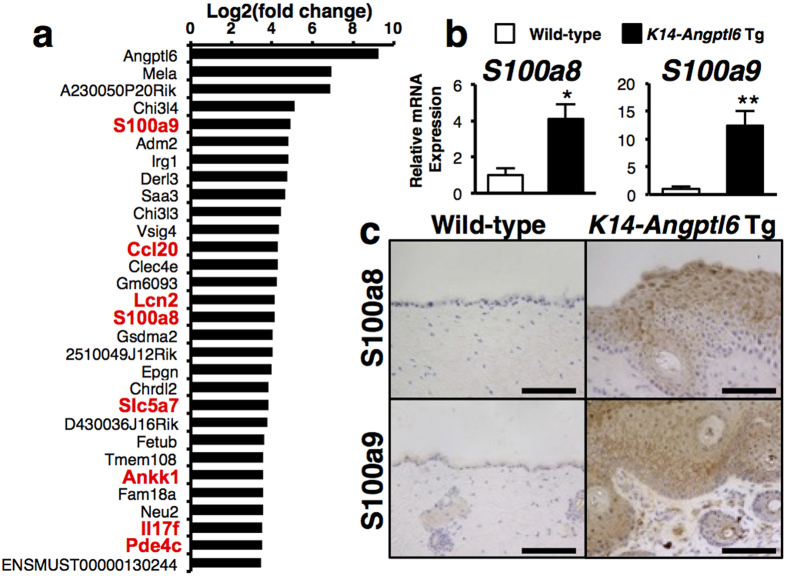
S100a8 and S100a9 levels increase in psoriasis-like lesions of K14-Angptl6 Tg mice. (**a**) The top 30-upregulated mRNAs in psoriatic-like lesions from 20 weeks of age of *K14-Angptl6* Tg relative to wild-type littermate mice. Genes shown in red were previously reported to be psoriatic-related genes^11^,^23^,^24^,^25^,^26^,^27^,^28^. (**b**) *S100a8* and *S100a9* transcript levels in psoriatic-like lesions of wild-type or *K14-Angptl6* Tg mice at 20 weeks of age. Values for respective WT mice were set to 1 (n = 4–5 per each group). Data are means ± SEM. *p < 0.05, **p < 0.01 between genotypes. (**c**) Immunohistochemistry staining for S100a8 and S100a9 in neck skin of WT and *K14-Angptl6* Tg mice at 20 weeks of age. Scale bar: 100 μm.

**Figure 3:**
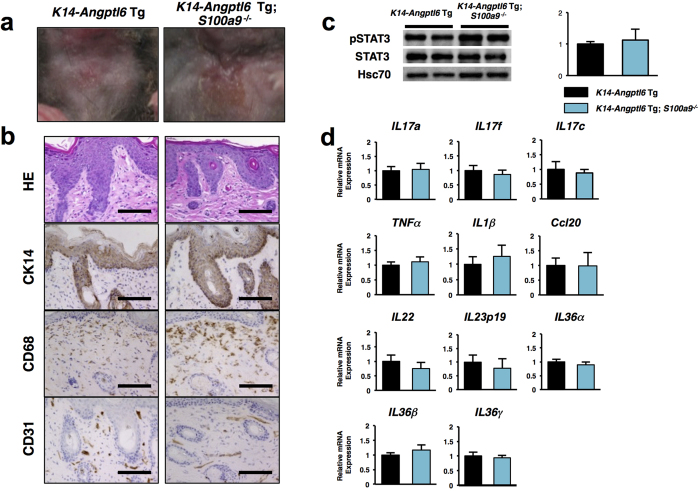
S100a9 loss in K14-Angptl6 Tg mice does not rescue psoriatic phenotypes. (**a**) Neck skin of *K14-Angptl6* Tg (left) and *K14-Angptl6* Tg;*S100a9*^−/−^ (right) mice at 20 weeks of age. Images are magnified from white-framed regions shown in Supplementary Fig. S3a. (**b**) HE staining and immunohistochemistry for CK14 (early keratinocyte marker), CD68 (macrophage marker) and CD31 (endothelial cell marker) in neck skin of *K14-Angptl6* Tg (left) and *K14-Angptl6* Tg;*S100a9*^−/−^ (right) mice. Images are magnified from black-framed regions shown in Supplementary Fig. S3b. Scale bar: 100 μm. (**c**) Representative immunoblotting and quantitation of ratio of p-STAT3 to total STAT3 from skin samples of *K14-Angptl6* Tg versus *K14-Angptl6* Tg;*S100a9*^−/−^ mice at 20 weeks of age. Hsc70 served as a loading control. Values from *K14-Angptl6* Tg mice were set to 1 (n = 3 per each group). (**d**) Quantitative real-time RT-PCR analysis of cytokine/chemokine expression in ear tissues of *K14-Angptl6* Tg and *K14-Angptl6* Tg;*S100a9*^−/−^ mice at 20 weeks of age. Values for respective *K14-Angptl6* Tg mice were set to 1 (n = 8–10 per each group). Data are means ± SEM.

To define molecular mechanisms underlying skin pathologies seen in *K14-Angptl6* Tg mice, we undertook gene-expression profiling of whole ear tissue from 20-week-old *K14-Angptl6* Tg versus comparably aged wild-type littermates using RNA sequencing technology (Fig. 2a, Tables 1 and 2). Of transcripts that increased in *K14-Angptl6* Tg mice, among the top 30 were several genes associated with psoriasis (Fig. 2a and Table 1)^11^,^23^,^24^,^25^,^26^,^27^,^28^. High levels of S100a8/S100a9 (also known as calprotectin) are reportedly induced in epidermal keratinocytes in wound healing or in inflammatory processes, including psoriasis, while normal epidermis shows very low levels^11^,^29^. We found that S100a8 and S100a9 transcript (Fig. 2b) and protein (Fig. 2c) levels increased in skin tissue of *K14-Angptl6* Tg relative to wild-type controls. Recent reports indicate that the absence of S100A8 protein was also detected in *S100a9*^−/−^ mice due to inefficient translation of *S100a8* mRNA or due to instability of S100A8 protein in the absence of its partner, S100A9^11^,^30^. Moreover, psoriasis-like skin proliferative and inflammatory phenotypes are significantly rescued in psoriasis model mice in which S100A9 is genetically deleted^11^, suggesting that S100A9 could be a useful therapeutic target for psoriasis. Therefore, we asked whether S100A9 loss would alter psoriasis-like skin phenotypes in *K14-Angptl6* Tg mice by genetically deleting *S100a9* in *K14-Angptl6* Tg mice (*K14-Angptl6* Tg;*S100a9*^−/−^) using CRISPR-Cas9-mediated technology (Supplementary Fig. S2). Overall, the gross appearance of skin in *K14-Angptl6* Tg;*S100a9*^−/−^ and *K14-Angptl6* Tg littermates exhibited indistinguishable inflammatory phenotypes throughout animal’s lifespan (Fig. 3a and Supplementary Figs S3a and S4a). Histological and immunohistochemical analysis of skin tissue from mice of both genotypes with antibodies to CK14, CD68 and CD31 (markers of early keratinocytes, macrophages, and endothelial cells, respectively) revealed no differences in pathology of psoriasis-like lesions (Fig. 3b and Supplementary Figs S3b and S4b). Moreover, the Koebner phenomenon in skin tissue promoted by tape stripping injury was observed in both *K14-Angptl6* Tg;*S100a9*^−/−^ and *K14-Angptl6* Tg mice (Supplementary Fig. S3c). Moreover, both genotypes showed comparable STAT3 activation and expression levels of psoriasis-associated chemokine/cytokines (Fig. 3c,d and Supplementary Figs S3d and S4c,d). Thus, S100A9 loss in *K14-Angptl6* Tg male or female mice did not rescue psoriasis-like skin phenotypes, suggesting that ANGPTL6-dependent psoriasis emerges independently of S100A9 and that these phenotypes are not sex-dependent.

### ANGPTL6 production increases in keratinocytes of some psoriasis patients

**Figure 4:**
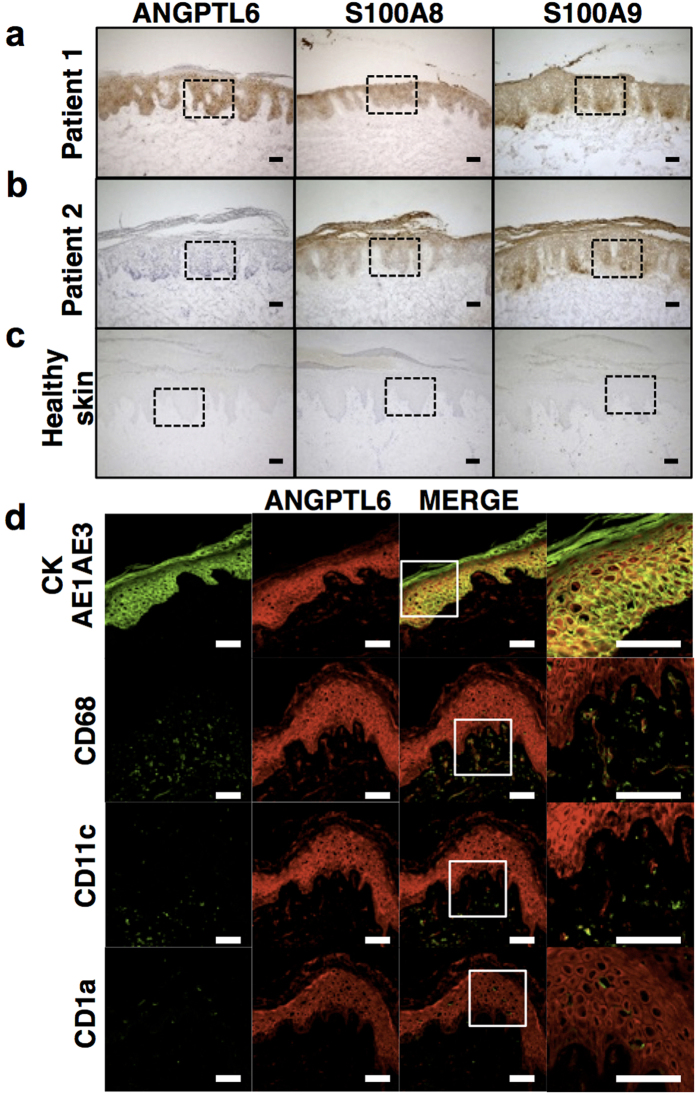
ANGPTL6 expression is elevated in human psoriatic skin. (**a**–**c**) Immunohistochemical staining of serial sections of human skin tissues with antibodies against ANGPTL6, S100A8 and S100A9. Shown are samples from two representative psoriasis patients, including (**a**) Patient 1 (37-year-old female (Case No. 2 in Table 3)) and (**b**) Patient 2 (37-year-old female (Case No.14 in Table 3)). (**c**) Also shown is a sample from a healthy control subject (34-year-old female (Case No. 2 in Table 4)). Magnified images of each dotted box are shown in Supplementary Fig. S5. Scale bar: 100 μm. (**d**) Representative confocal images from double immunofluorescence staining for AE1AE3 (keratinocyte marker; green) and ANGPTL6 (red, top row); CD68 (macrophage marker; green) and ANGPTL6 (red, second row); CD11c (dendritic cell marker; green) and ANGPTL6 (red, third row); and CD1a (Langerhans cell marker; green) and ANGPTL6 (red, bottom row) of a human psoriatic lesion (56-year-old male (Case No. 10 in Table 3)). Scale bar: 100 μm. Right column shows magnified images of boxed regions in adjacent image. Scale bar: 50 μm.

Finally, we asked whether ANGPTL6 activation is correlated with skin tissue pathologies seen in psoriasis patients. To do so, we compared ANGPTL6 protein levels between psoriasis patients and non-psoriasis control subjects (Tables 3 and 4). Immunohistochemical analysis of tissue specimens with ANGPTL6 antibody revealed abundant or moderate ANGPTL6 protein in the epidermal layer of 13 of 15 tissue specimens from different patients, whereas no ANGPTL6 protein was detected in all 10 non-psoriasis control specimens (Fig. 4a,c and Supplementary Fig. S5a,c). Two patients did not show elevated ANGPTL6 production in skin tissue (Fig. 4b and Supplementary Fig. S5b). Immunofluorescence analysis of skin tissue from one psoriasis patient revealed ANGPTL6 production in epidermal keratinocytes, suggesting they are the source of ANGPTL6 in skin tissue of these patients (Fig. 4d). By contrast, S100A8 and S100A9 proteins were abundant in skin tissues of all 15 psoriasis patients but not in tissues from any non-psoriasis control subject (Fig. 4, Supplementary Fig. S5, Tables 3 and 4), suggesting that ANGPTL6 production and S100A8/S100A9 expression are not linked in the pathogenesis of psoriasis. Thus, elevated ANGPTL6 levels seen in keratinocytes of psoriasis patients support our observations in mice that ANGPTL6 may induce psoriasis-like phenotypes.

## Discussion

Over the past decades, various mouse models of skin pathologies resembling psoriasis have provided important insight into molecular mechanisms undelying psoriasis^3^. Nonetheless, differences in immune-associated gene expression between mouse models and psoriatic patients^31^ require development of additional models that reflect unique skin phenotypes and gene expression patterns seen in patients. In this study, we report that *K14-Angptl6* Tg mice develop psoriasis-like epidermal proliferation consisting of prematurely differentiated keratinocytes, which contributes to epidermal barrier dysfunction, and also exhibit psoriasis-like skin tissue inflammation marked by infiltration of neutrophils and vascular endothelial cells into the dermal layer. We also observed enhanced STAT3 activation in skin tissue of transgenics and upregulated expression of psoriasis-associated chemokines/cytokines. Finally, we report that ANGPTL6 production is elevated in keratinocytes from some psoriasis patients. Our results strongly suggest that increased ANGPTL6 production from keratinocytes contributes to psoriasis pathogenesis and that *K14-Angptl6* Tg mice could serve as alternate mouse models of the disease. They also suggest that *K14-Angptl6* Tg mice could serve as a murine psoriatic model for preclinical testing of biologics useful to attenuate the condition.

Macrophages reportedly play an important role in the pathophysiological development of psoriasis, and their deletion reportedly can rescue psoriasis skin phenotypes^32^,^33^. In this study, we observed that infiltrating macrophages express ANGPTL6 in response to injury of normal mouse skin. Thus, deletion of macrophages via clodronate liposome treatment may rescue ANGPTL6-dependent psoriasis when skin tissues repeatedly suffer injurious stimuli. In regard to skin tissue repair after injury, however, ANGPTL6 may function in keratinocyte proliferation. Further studies are needed to investigate mechanisms by which ANGPTL6 promotes not only psoriasis development but also skin tissue repair in physiological conditions in response to normal skin injury.

Psoriasis starts with angiogenesis in the superficial dermal microvasculature^34^. Psoriatic skin is characterized by increased expansion and permeability of the endothelium of the superficial microvasculature^35^. These phenotypes allow leukocyte transmigration into areas of inflammation via enhanced expression of cell adhesion molecules associated with the onset of psoriasis^36^,^37^. We previously reported that ANGPTL6 induces not only neovascularization but also enhanced permeability of the local vasculature^16^. These data suggest that ANGPTL6 modulates permeability of the microvasculature and/or immature neomicrovasculature and accelerates leukocyte transmigration into dermal areas at the onset of inflammation.

Although human psoriasis has a multifactorial etiology, it is strongly influenced by genetics^2^. A search for genes responsible for familial psoriasis via genome-wide association study (GWAS) identified several putative psoriasis susceptibility loci^38^. Interestingly, human *ANGPTL6* is located at 19p13.2, located in the psoriasis susceptibility region *PSORS6*^14^,^15^. It would be of interest to investigate whether ANGPTL6 levels are elevated in skin tissues of individuals with mutations in *PSORS6.* Also, a recent report suggests significant association of *ANGPTL6* gene polymorphisms (rs6511435) tends to be associated with a 20% higher risk of metabolic syndrome^39^, while another found that serum ANGPTL6 levels are significantly higher in subjects with metabolic syndrome than in healthy individuals^40^. Interestingly, psoriasis patients have a higher prevalence of metabolic syndrome^41^,^42^; thus it would also be of interest to investigate whether there are ANGPTL6 gene polymorphisms associated with psoriasis.

Our gene expression profile analysis of *K14-Angptl6* Tg mice revealed several genes whose expression levels were previously reported to increase in psoriasis skin tissues^11^,^23^,^24^,^25^,^26^,^27^,^28^. One of those was S100A9, which was recently suggested to be a potential therapeutic target for psoriasis^11^. Interestingly, psoriasis-like skin proliferative and inflammatory phenotypes were not rescued in *K14-Angptl6* Tg mice by S100A9 deletion. This observation is consistent with our analysis of patient specimens: we observed elevated ANGPTL6 levels in keratinocytes of some psoriasis patients, while S100A8 and S100A9 were elevated in keratinocytes of all patients. We suggest that ANGPTL6-dependent mechanisms associated with psoriasis may be independent of those governed by S100A9. Our gene profiling analysis also revealed *A230050P20Rik* to be one of the most significantly upregulated transcripts in *K14-Angptl6* Tg mice. Interestingly, a recent paper showed that chromosome 19 open reading frame 66 (C19orf66), the human ortholog of *A230050P20Rik*, functions as an interferon-stimulated cellular inhibitor of dengue virus replication^43^. Moreover, the Gene Expression Omnibus (GEO, National Center for Biotechnology Information (NCBI) database (http://www.ncbi.nlm.nih.gov/geoprofiles/) shows that C19orf66 expression is elevated in human psoriatic skin relative to normal tissue (database sets of GDS4602 and GDS4891). Future studies should address whether C19orf66 functions in development of psoriatic skin lesions.

In summary, ours is the first study to report that in mice, epidermal ANGPTL6 activity promotes aberrant epidermal barrier function associated with keratinocyte hyperproliferation and hyperactive chemokine/cytokine signaling, and is accompanied by neutrophil infiltration and concomitant release of proinflammatory factors in skin lesions. We also found that infiltrated macrophage-derived ANGPTL6 is upregulated by physical stimuli, such as skin injury. We conclude that *K14-Angptl6* Tg mice represent a novel symptomatic, histological, and molecular model of the condition and could be useful in preclinical testing of new therapeutics against psoriasis.

## Methods

### Animal study

For this study, 20-week-old male and female wild-type mice on a C57BL/6N genetic background (Kyudo Co., Ltd., Saga, Japan), transgenic mice expressing Angptl6 in keratinocytes (*K14-Angptl6* Tg)^13^, and S100a9-knockout (*S100a9*^−/−^) mice, plus respective wild-type littermates on a C57BL/6N background were used. All animals were fed a normal diet (CE-2, CLEA, Tokyo, Japan), bred in a mouse house under specific pathogen-free (SPF) conditions with automatically controlled lighting (12 h on, 12 h off), and maintained at a stable temperature of 23 °C in the Animal Resource Facility at Kumamoto University. Tape stripping analysis was performed using 20 strokes with transparent tape on dorsal skin of 10- to 12-week-old mice following hair removal, under sodium pentobarbital anesthesia. TEWL of mouse skin was determined using a Tewameter TM300 (Courage and Khazaka, Cologne, Germany) as described^44^. All the experimental procedures were approved by the Committee on Animal Research at Kumamoto University and conducted in accordance with guidelines of Institutional Animal Committee of Kumamoto University.

### Immunohistochemical staining

Human and mouse skin tissues were embedded in OCT compound (Sakura Finetechnical, Tokyo, Japan) and frozen in liquid nitrogen. Mouse skin tissues were fixed in 4% paraformaldehyde for 24 h and embedded in paraffin. Hematoxylin and eosin (HE) was used to stain 6 μm-thick frozen sections and 4-μm-thick paraffinized sections. For immunohistochemistry, a rabbit polyclonal anti-human ANGPTL6 antibodies (1:100) was produced by immunizing rabbits with a synthetic peptide corresponding to amino acids 392–408 (NDKPESTVDRDRDSYSG) of ANGPTL6, and a rabbit polyclonal anti-mouse Angptl6 (1:100) was used previously reported^13^. Supplementary Table S2 shows information relevant to other primary antibodies. Fixed sections from back, neck and ear skin of *K14-Angptl6* Tg mice and their littermate wild-type controls were stained with 1:500 diluted anti-mouse Angptl6 antibody. Sections were pretreated with periodic acid (Nichirei, Tokyo, Japan) to inhibit endogenous peroxidases. Subsequently, specimens were incubated overnight with each antibody dilution. After washing sections with phosphate-buffered saline (PBS), immunostaining was performed using Histofine rat stain kit, Histofine goat stain kit (Nichirei Biosciences Inc), or EnVision/horseradish peroxidase (Dako) according to the manufacturers’ instruction, and specimens were then counterstained with hematoxylin. As negative controls, the same procedures were performed using isotype control IgG rather than primary antibodies. Peroxidase activity was visualized by incubation with a 3,3-diaminobenzidine solution and analysis using a BIOREVO BZ-9000 microscope (Keyence, Osaka, Japan). For double immunofluorescence, a rabbit polyclonal anti-human ANGPTL6 antibody (1:100) was used with other primary antibodies and Alexa Fluor-conjugated secondary antibodies, as indicated in Supplementary Table S2. After washing sections with PBS, fluorescent images were captured by confocal laser microscopy (FluoView FV1200, Olympus Corporation, Japan).

### Western blot analysis

Mouse skin tissue was homogenized in RIPA buffer (1% NP-40, 50 mM Tris-HCl (pH7.5), 150 mM NaCl, 0.1% SDS, 0.5% sodium deoxycholate, 5 mM NaF, 1 mM EDTA, 1 mM Na_3_VO_4_, plus a protease inhibitor cocktail [Nacalai Tesque, Kyoto Japan]). Total protein (10 μg) was separated by SDS-PAGE and transferred to PVDF membranes. For Angptl6 immunoblotting, membranes were reacted with 1:1000 diluted polyclonal rabbit anti-mouse Angptl6 antibodies, 1:1000 diluted polyclonal rabbit anti-mouse S100A8 antibody (sc-8112; SantaCruz Biotechnology, SantaCruz, CA), 1:1000 diluted polyclonal rabbit anti-mouse S100A9 antibody (sc-8115; SantaCruz Biotechnology, SantaCruz, CA), 1:2000 diluted monoclonal rabbit anti-Phospho-STAT3 antibody (9145S; Cell Signaling Technology Japan, Tokyo, Japan), or 1:2000 diluted monoclonal rabbit anti-STAT3 antibody (4904S; Cell Signaling Technology Japan, Tokyo, Japan) overnight at 4 °C. After PBS or TBS washing, membranes were reacted with 1:2000 diluted streptavidin-horseradish peroxidase (HRP)-conjugated secondary antibody (Thermo Fisher Scientific, Waltham, MA) at room temperature for 60 min. As an internal control, 1:2000 diluted mouse anti-Hsc70 (sc-7298; SantaCruz Biotechnology, Santa Cruz, CA) and 1:3000 diluted HRP-conjugated sheep anti-mouse IgG (Amersham Pharmatech Biotech, Piscataway, NJ) antibodies served as first and secondary antibodies, respectively. Reacted membranes were visualized using ECL Western Blotting Detection Reagent (GE Healthcare, Little Chalfont, UK). Band intensities were quantified utilizing image-analyzing software (Image J, NIH, Bethesda, MD).

### Real-time quantitative RT-PCR analysis

Total RNA isolation, cDNA synthesis, and quantitative RT-PCR were performed as described^45^. Relative transcript abundance was normalized to that of *β-actin* level. PCR oligonucleotides are listed in Supplementary Table S1.

### RNA sequencing

RNA sequencing was performed as described^46^. In brief, sample libraries established from whole ear tissues of 20-week-old *K14-Angptl6* Tg or littermate wild-type mice were prepared using a TruSeq RNA Sample Prep Kit (Illumina, San Diego, CA). Sequencing runs were performed on an Illumina Genome Analyzer IIx (Illumina, San Diego, CA). To evaluate differential gene expression, expression data was normalized and gene annotations were added using RegionMiner with Genomatix Genome Analyzer (Genomatix, Munich, Germany) software. To compile NE-value information for an individual gene, mean NE-values were calculated from all gene transcripts, as were log2-transformed fold-changes in ear tissues of *K14-Angptl6* Tg versus wild-type littermates. RNA-seq data were deposited in the DDBJ Sequence Read Archive (DRA). Accession number: DRA004512.

### Generation of *S100a9* null mice

Target regions for *S100a9* gene disruption by CRISPR-Cas9-mediated cleavage were identified as 23 nt sequences harboring the canonical “NGG” proto-spacer adjacent motif (PAM) and a unique 5′ sequence unrelated to other genomic sequences. Two single guide RNAs (sgRNAs) that targeted opposing exon 2 DNA strands including the *S100a9* initiator ATG were designed (Supplementary Fig. S2a). Then gene targeting of *S100a9* in fertilized eggs of *K14-Angptl6* Tg mice was undertaken using DNA double-stranded break methods and the CRISPR/Cas9 genome editing system with pX330 (http://www.addgene.org/42230/)^47^. Genotyping of tail DNA was performed using the following primers: S100a9-S (5′-CTTCCTTCACTTTTTCTAAG-3′) and S100a9-AS (5′-CTGGGATATGGGTTCCTAG-3′) (Supplementary Fig. S2b). Wild-type and mutant alleles yielded 614 bp and 308 bp bands, respectively. S100a9 nulls were confirmed by semi-qRT-PCR using bone marrow tissue and the following primers: S100a9-For (5′-GGGCTTACACTGCTCTTACC-3′) and S100a9-Rev (5′-AGTCATGGCTGCCTCCAGAGA-3′) and G3PDH control primers (Toyobo, Japan)(Supplementary Fig. S2c). The level of *S100a9* transcript was also confirmed by qRT-PCR using bone marrow tissue and skin using *S100a9* and *β-Actin* PCR oligonucleotides, as listed in Supplementary Table S1 (Supplementary Fig. S2d). *S100a9*^−/−^ male and female mice were viable and exhibited no gross skin phenotypes, in accord with previous reports (Supplementary Fig. S3a)^30^.

### Human skin samples

Human skin biopsies of psoriatic lesions from 15 outpatients (aged 42.2 ± 2.7 years; 10 males, 5 females; Table 3) were obtained. Ten biopsies of healthy skin derived from regions adjacent to pigmented nevi served as control tissues (patients were aged 31.6 ± 3.8 years; 3males, 7 females; Table 4). Specimens were acquired at Kumamoto University Hospital between May of 2013 and Dec of 2015. Institutional review board approval and written informed consent was obtained from each outpatient and normal subjects were entered into this study according to the Declaration of Helsinki.

### Statistical analysis

All values were reported as the mean ± s.e.m. Data were assessed as two-group comparisons of variables by unpaired and paired two-tailed *t*-tests. *P-*values < 0.05 was considered statistically significant.

## Additional Information

**How to cite this article**: Tanigawa, H. *et al*. Upregulation of ANGPTL6 in mouse keratinocytes enhances susceptibility to psoriasis. *Sci. Rep.* **6**, 34690; doi: 10.1038/srep34690 (2016).

## Supplementary Material

Supplementary Information

## Figures and Tables

**Table 1:** The top 30-upregulated mRNAs in psoriatic-like lesions from 20-week-old *K14-Angptl6* Tg relative to wild-type littermate mice.

	Symbol	log_2_ (fold change)	p-value
1	*Angptl6*	9.26	<0.001
2	*Mela*	6.94	<0.001
3	*A230050P20Rik*	6.87	<0.001
4	*Chi3l4*	5.12	<0.001
5	*S100a9*^(11)^	4.9	<0.001
6	*Adm2*	4.81	<0.001
7	*Irg1*	4.81	<0.001
8	*Derl3*	4.73	<0.001
9	*Saa3*	4.67	<0.001
10	*Chi3l3*	4.45	<0.001
11	*Vsig4*	4.32	<0.001
12	*Ccl20*^(23)^	4.31	<0.001
13	*Clec4e*	4.28	<0.001
14	*Gm6093*	4.24	<0.001
15	*Lcn2*^(24)^	4.15	<0.001
16	*S100a8*^(11)^	4.13	<0.001
17	*Gsdma2*	4.05	<0.001
18	*2510049J12Rik*	4.02	<0.001
19	*Epgn*	3.99	<0.001
20	*Chrdl2*	3.84	<0.001
21	*Slc5a7*^(25)^	3.81	<0.001
22	*D430036J16Rik*	3.75	<0.001
23	*Fetub*	3.62	<0.001
24	*Tmem108*	3.58	<0.001
25	*Ankk1*^(26)^	3.54	<0.001
26	*Fam18a*	3.54	<0.001
27	*Neu2*	3.54	<0.001
28	*Il17f *^(27)^	3.52	<0.001
29	*Pde4c*^(28)^	3.52	<0.005
30	*ENSMUST00000130244*	3.47	<0.001

**Table 2:** The 30 most significantly downregulated mRNAs in psoriatic-like lesions from 20-week-old *K14-Angptl6* Tg relative to wild-type littermate mice.

	Symbol	log_2_ (fold change)	p-value
1	*Klrk1*	−8.63	<0.001
2	*Srgap1*	−5.77	<0.005
3	*Tmem165*	−5.22	<0.001
4	*LOC100502803*	−5.08	<0.01
5	*Mx1*	−4.87	<0.001
6	*Kcnh6*	−4.75	<0.001
7	*Pyy*	−4.59	<0.001
8	*Gm11596*	−4.34	<0.001
9	*Cyp2a5*	−4.23	<0.001
10	*LOC16697*	−4.23	<0.001
11	*Gm11555*	−4.20	<0.001
12	*Muc4*	−4.09	<0.001
13	*Paqr7*	−4.07	<0.001
14	*Wdr4*	−4.06	<0.001
15	*Alox15*	−3.99	<0.001
16	*Gm4553*	−3.99	<0.001
17	*Klri2*	−3.95	<0.001
18	*Krt74*	−3.94	<0.001
19	*1110032F04Rik*	−3.91	<0.001
20	*AK044745*	−3.91	<0.005
21	*AK162617*	−3.91	<0.001
22	*D830012I16Rik*	−3.91	<0.001
23	*Gm2696*	−3.89	<0.001
24	*Krtap4-16*	−3.86	<0.001
25	*Sftpb*	−3.86	<0.001
26	*Gjd3*	−3.83	<0.001
27	*5033406O09Rik*	−3.81	<0.001
28	*AK042705*	−3.81	<0.001
29	*Tsks*	−3.81	<0.001
30	*Ltbp1*	−3.74	<0.001

**Table 3:** ANGPTL6, S100A8 and S100A9 expression in skin biopsies from psoriasis patients.

Case No.	Age	Sex	ANGPTL6 Expression	S100A8 Expression	S100A9 Expression
1	53	M	(++)	(++)	(+)
2	37	F	(++)	(++)	(++)
3	45	M	(++)	(++)	(++)
4	43	M	(++)	(++)	(++)
5	27	M	(+)	(++)	(+)
6	56	M	(+)	(+)	(++)
7	46	M	(+)	(++)	(++)
8	40	M	(+)	(+)	(++)
9	55	M	(+)	(+)	(+)
10	56	M	(+)	(++)	(++)
11	25	F	(+)	(++)	(+)
12	41	M	(+)	(++)	(++)
13	28	F	(+)	(++)	(++)
14	37	F	(−)	(+)	(++)
15	44	F	(−)	(+)	(++)

**Table 4:** ANGPTL6, S100A8 and S100A9 expression in skin biopsies from healthy control subjects.

Case No.	Age	Sex	ANGPTL6 Expression	S100A8 Expression	S100A9 Expression
1	49	F	(−)	(−)	(−)
2	34	F	(−)	(−)	(−)
3	16	F	(−)	(−)	(+)
4	33	F	(−)	(−)	(−)
5	50	F	(−)	(−)	(−)
6	36	F	(−)	(−)	(−)
7	16	F	(−)	(−)	(−)
8	34	M	(−)	(−)	(−)
9	24	M	(−)	(−)	(−)
10	24	M	(−)	(−)	(−)
