# Supplementary material for: Upregulation of ANGPTL6 in mouse keratinocytes enhances susceptibility to psoriasis
Source: Sci Rep. 2016 Oct 4;6:34690. doi: 10.1038/srep34690 (PMC5048131; doi:10.1038/srep34690)
Supplement: Supplementary Information [file srep34690-s1.pdf]

Supplementary Information

Upregulation of ANGPTL6 in mouse keratinocytes enhances susceptibility to psoriasis

Hiroki Tanigawa, Keishi Miyata, Zhe Tian, Jun Aoi, Tsuyoshi Kadomatu, Satoshi Fukushima, Aki Ogata, Naoki Takeda, Jiabin Zhao, Shunshun Zhu, Kazutoyo Terada, Motoyoshi Endo, Jun Morinaga, Taichi Sugizaki, Michio Sato, Masaki Suimye Morioka, Ichiro Manabe, Youichi Mashimo, Akira Hata, Yoshitaka Taketomi, Kei Yamamoto, Makoto Murakami, Kimi Araki, Masatoshi Jinnin, Hironobu Ihn, and Yuichi Oike.

Supplementary Table S1.
Primer sequences used in quantitative RT-PCR

Gene		Sequences
β-actin	Forward	CATCCGTAAAGACCTCTATGCCAAC
	Reverse	ATGGAGCCACCGATCCACA
Ccl20	Forward	TGCTCTTCCTTGCTTTGGCATGGGTA
	Reverse	TCTGTGCAGTGATGTGCAGGTGAAGC
IL1β	Forward	TCCAGGATGAGGACATGAGCAC
	Reverse	GAACGTCACACACCAGCAGGTTA
IL17a	Forward	ACGCGCAAACATGAGTCCAG
	Reverse	AGGCTCAGCAGCAGCAACAG
IL17f	Forward	CCCATGGGATTACAACATCACTC
	Reverse	CACTGGGCCTCAGCGATC
IL17c	Forward	CCTCTAGCTGGAACACAGTGC
	Reverse	GCGGTTCTCATCTGTGTCG
IL22	Forward	ATACATCGTCAACCGCACCTTT
	Reverse	AGCCGGACATCTGTGTTGTTAT
IL23p19	Forward	AGCGGGACATATGAATCTACTAAGAGA
	Reverse	GTCCTAGTAGGGAGGTGTGAAGTTG
IL36α	Forward	TGCCCACTCATTCTGACCCA
	Reverse	GTGCCACAGAGCAATGTGTC
IL36β	Forward	ACAAAAAGCCTTTCTGTTCTATCAT
	Reverse	CCATGTTGGATTTACTTCTCAGACT
IL36γ	Forward	ATGGACACCCTACTTTGCTG
	Reverse	TGTCCGGGTGTGGTAAAACA
TNFα	Forward	AAGCCTGTAGCCCACGTCGTA
	Reverse	GGCACCACTAGTTGGTTGTCTTTG
S100a8	Forward	TGCGATGGTGATAAAAGTGG
	Reverse	GGCCAGAAGCTCTGCTACTC
S100a9	Forward	CACCCTGAGCAAGAAGGAAT
	Reverse	TGTCATTTATGAGGGCTTCATTT
Angptl6	Forward	CTTCACCAACTGGCAGCACTACA
	Reverse	CTAGGAGTATCAGCAGCTCGTGGTC

Supplementary Table S2.
Antibodies and dilutions used in this study

Antibody name	Company	Catalog number	Dilution
anti-mouse CK14 (rabbit)	BioLegend	PRB 155P-100	1:100
anti-mouse CD68 (rat)	Bio-Rad	MCA1957	1:100
anti-mouse CD31 (rat)	BD Biosciences	557355	1:100
anit-mouse Ly-6G (rat)	abcam	ab25377	1:100
anti-mouse S100a8 (goat)	SantaCruz Biotechnology	sc-8112	1:100
anti-mouse S100a9 (rabbit)	Novus Biologicals	NB110-89726	1:100
anti-mouse Neutrophil (rat)	abcam	ab2557	1:100
anti-mouse Mac3 (rat)	BD Biosciences	550292	1:100
anti-mouse CD4 (rat)	BD Biosciences	550278	1:100
anti-mouse CD45R/B220 (rat)	BioLedend	103201	1:200
anti-human Cytokeratin (mouse)	DAKO	IR053	1:100
anti-human CD68 (mouse)	DAKO	M0876	1:100
anti-human CD11c (mouse)	BD Biosciences	550375	1:20
anti-human CD1a (mouse)	Immunotech	1590	1:100
anti-human S100A8 (mouse)	abcam	ab19860	1:200
anti-human S100A9 (mouse)	Acris	BM4027	1:200
Alexa Fluor488-conjugated anti-rat antibody (donkey)	Thermo Fisher Scientific	A21208	1:500
Alexa Fluor488-conjugated anti-mouse antibody (donkey)	Thermo Fisher Scientific	A21202	1:500
Alexa Fluor594-conjugated anti-rabbit antibody (donkey)	Thermo Fisher Scientific	A21207	1:500

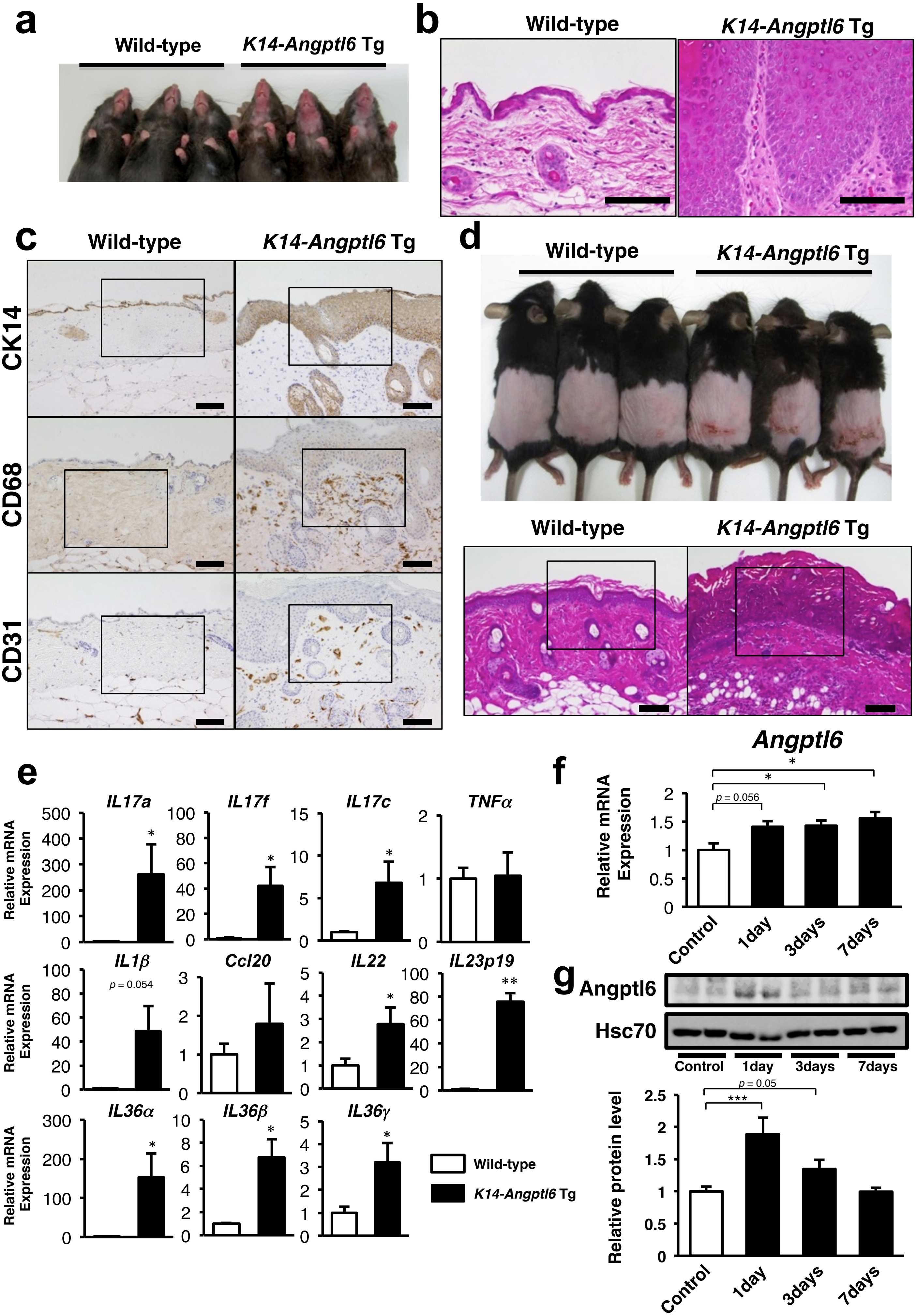

Supplementary Figure S1. Tanigawa *et al.*

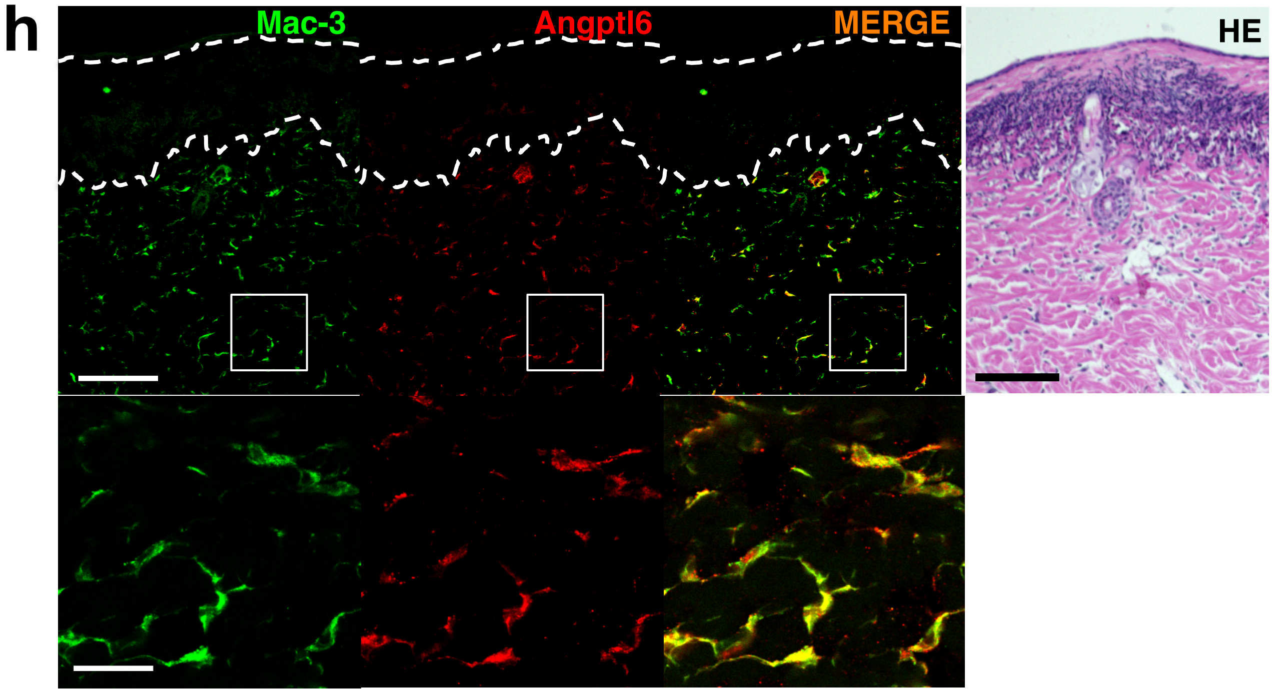

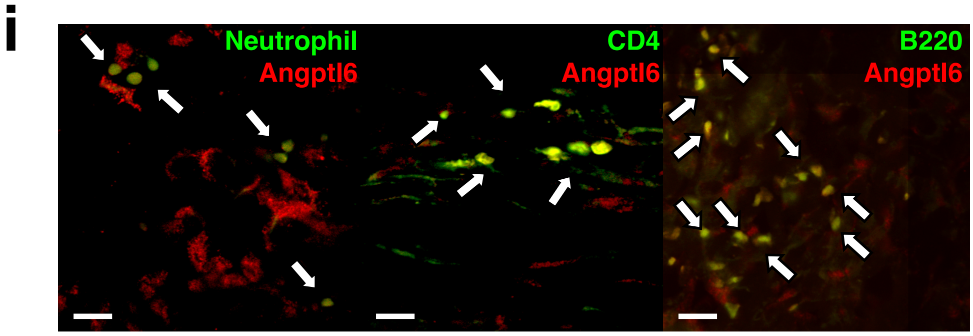

Supplementary Figure S1: Quantitative real-time RT-PCR analysis of psoriasis-related cytokine transcripts in neck skin of K14-Angptl6 Tg and wild-type littermate mice (**a**) *K14-Angptl6* Tg mice spontaneously develop psoriasiform skin by 20 weeks of age. A magnified image is shown in Fig. 1a. (**b**) HE-stained neck skin of wild-type (left) and *K14-Angptl6* Tg (right) 20-week-old mice. Images are magnified from black-dotted boxes in Fig. 1b. Scale bar: 100μm (**c**) CK14 (early keratinocyte marker), CD68 (macrophage marker) and CD31 (endothelial cell marker) staining of neck skin of wild-type (left) and *K14-Angptl6* Tg (right) 20-week-old mice. Corresponding magnified images of black-framed regions are shown in Fig. 1c. Scale bar: 100μm. (**d**) Assessment of potential lesions in wild-type and *K14-Angptl6* Tg mice, 4 days after tape stripping (upper), and HE-stained sections of those lesions (lower). Magnifications of black-framed regions are shown in Fig. 1d. Scale bar: 100μm. (**e**) Quantitative real-time RT-PCR analyses of indicated transcripts in neck skin. Values for respective WT mice were set to 1 (n = 7-8 per each group). (**f**) Angptl6 mRNA expression in back skin of wild-control mouse controls at 12 weeks and at indicated days after tape stripping procedure. Values for respective control mice were set to 1 (n = 5-6 per each group). Data are means ± SEM. **p* < 0.05, ***p* < 0.01 between genotypes or groups. (**g**) Representative immunoblotting and quantitation of ratio of Angptl6 to Hsc70 from back skin samples of wild-type mouse at indicated days after tape stripping procedure. Hsc70 served as a loading control. Values for control mouse were set to 1 (n = 5 per each group). (**h**) Representative confocal images from double immunofluorescence staining for Mac-3 (macrophage marker; green) and Angptl6 (red) and HE staining of a mouse skin 1 day after tape stripping. Scale bar: 100 μm (upper). Dotted lines indicate the border epidermis and dermis. Bottom column shows magnified images of boxed regions in adjacent image. Scale bar: 10 μm. (**i**) Representative confocal images from double immunofluorescence staining for Neutrophil (green), CD4 (T cell marker; green), B220 (B cell marker; green) and Angptl6 (red) of a mouse skin 1 day after tape stripping. Arrows indicate double-labeled cells. Scale bar: 10 μm.

Supplementary Figure S1. Tanigawa *et al.*

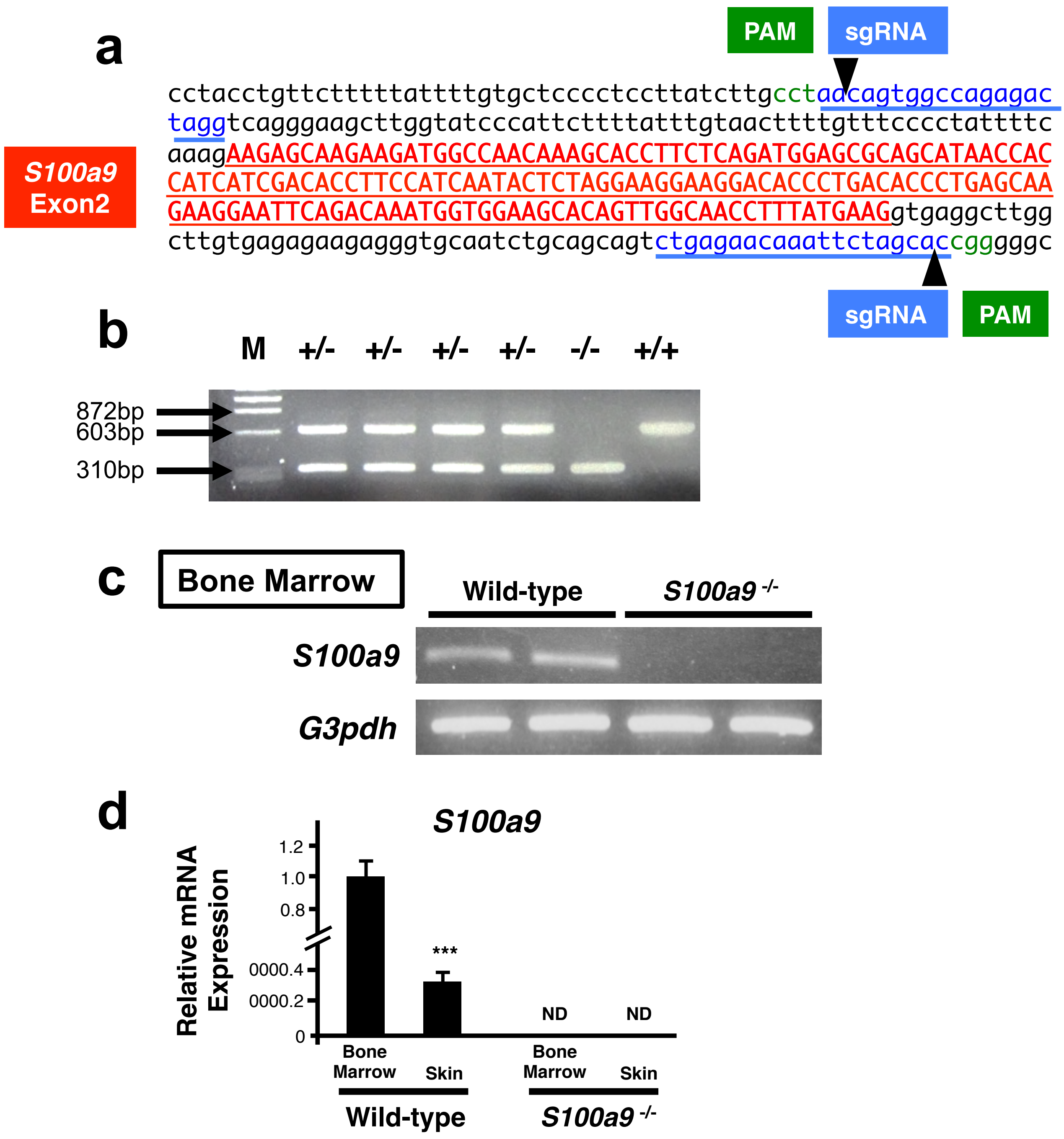

Supplementary Figure S2: Generation of *S100a9*$^{-/-}$ mice using the CRISPR/Cas9 system

(**a**) Sequence of mouse S100A9 and of primers used for CRISPER/Cas9 procedure. Indicated are proto-spacer adjacent motif (PAM) (green), single guide RNA (sgRNA) (blue), cleavage sites (black triangles), and the mouse *S100a9* second exon including the ATG (red capital letters). (**b**) Genotyping analysis of indicated genomic DNAs from mouse bone barrow cells. M: DNA size marker. A 614bp band from the wild-type allele is lost in null mice. (**c**) RT-PCR expression analysis of *S100a9* and *G3PDH* (as loading control) transcripts in bone marrow cells of wild-type and *S100a9*$^{-/-}$ mice. (**d**) *S100a9* mRNA expression analysis in bone marrow cells and back skin of wild-type and *S100a9*$^{-/-}$ mice at 12 weeks. Values for bone marrow cells of wild-type mice were set to 1 (n = 4 per each group). Data are means ± SEM. ***p < 0.001 between groups. ND; no detectable.

Supplementary Figure S2. Tanigawa *et al.*

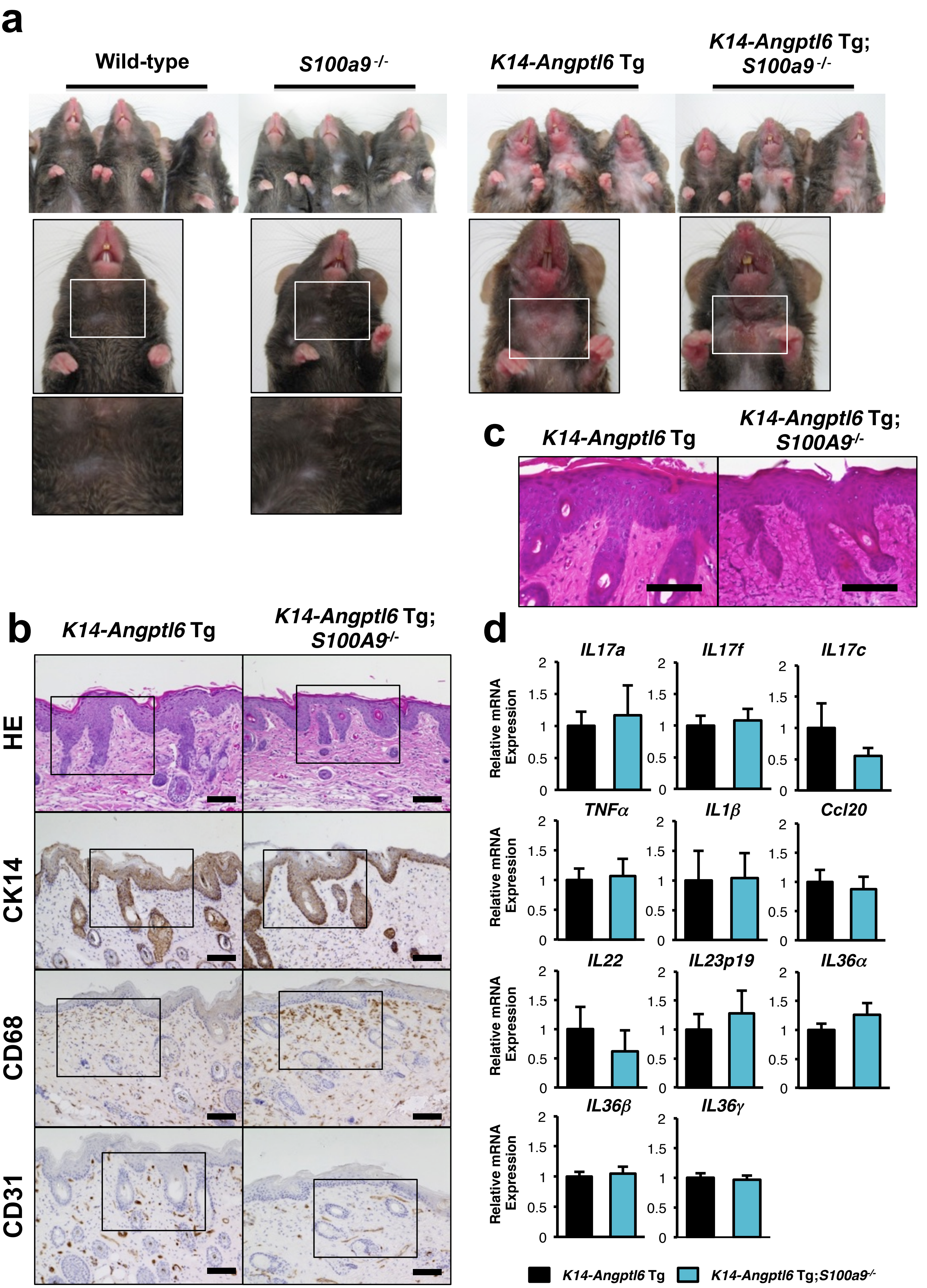

Supplementary Figure S3. Tanigawa *et al.*

Supplementary Figure S3: *K14-Angptl6* Tg;*S100a9*$^{-/-}$ and *K14-Angptl6* Tg male mice show comparable phenotypes

(**a**) (Left) Image showing neck skin of wild-type and *S100a9*$^{-/-}$ 20-week-old male mice (upper). Magnifications of corresponding regions within white frames are shown in bottom panels. (**Right**) Image showing neck skin of *K14-Angptl6* Tg and *K14-Angptl6* Tg;*S100a9*$^{-/-}$ 20-week-old male mice (upper). Magnified images of corresponding regions in white frames are shown in Fig. 3a. (**b**) HE staining and CK14 (early keratinocyte marker), CD68 (macrophage marker) and CD31 (endothelial cell marker) staining of neck skin from *K14-Angptl6* Tg (left) and *K14-Angptl6* Tg;*S100a9*$^{-/-}$ (right) male mice. Magnified images of corresponding black-framed regions are shown in Fig. 3b. Scale bar: 100μm. (**c**) HE-stained skin tissues after tape stripping procedure in *K14-Angptl6* Tg (left) and *K14-Angptl6* Tg;*S100a9*$^{-/-}$ (right) male mice. Scale bar: 100μm. (**d**) Quantitative real-time RT-PCR analyses of indicated transcripts in neck skin samples. Values for respective *K14-Angptl6* Tg male mice were set to 1(n = 7-8 per each group). Data are means ± SEM. *$p < 0.05$, **$p < 0.01$ between genotypes.

Supplementary Figure S3. Tanigawa *et al.*

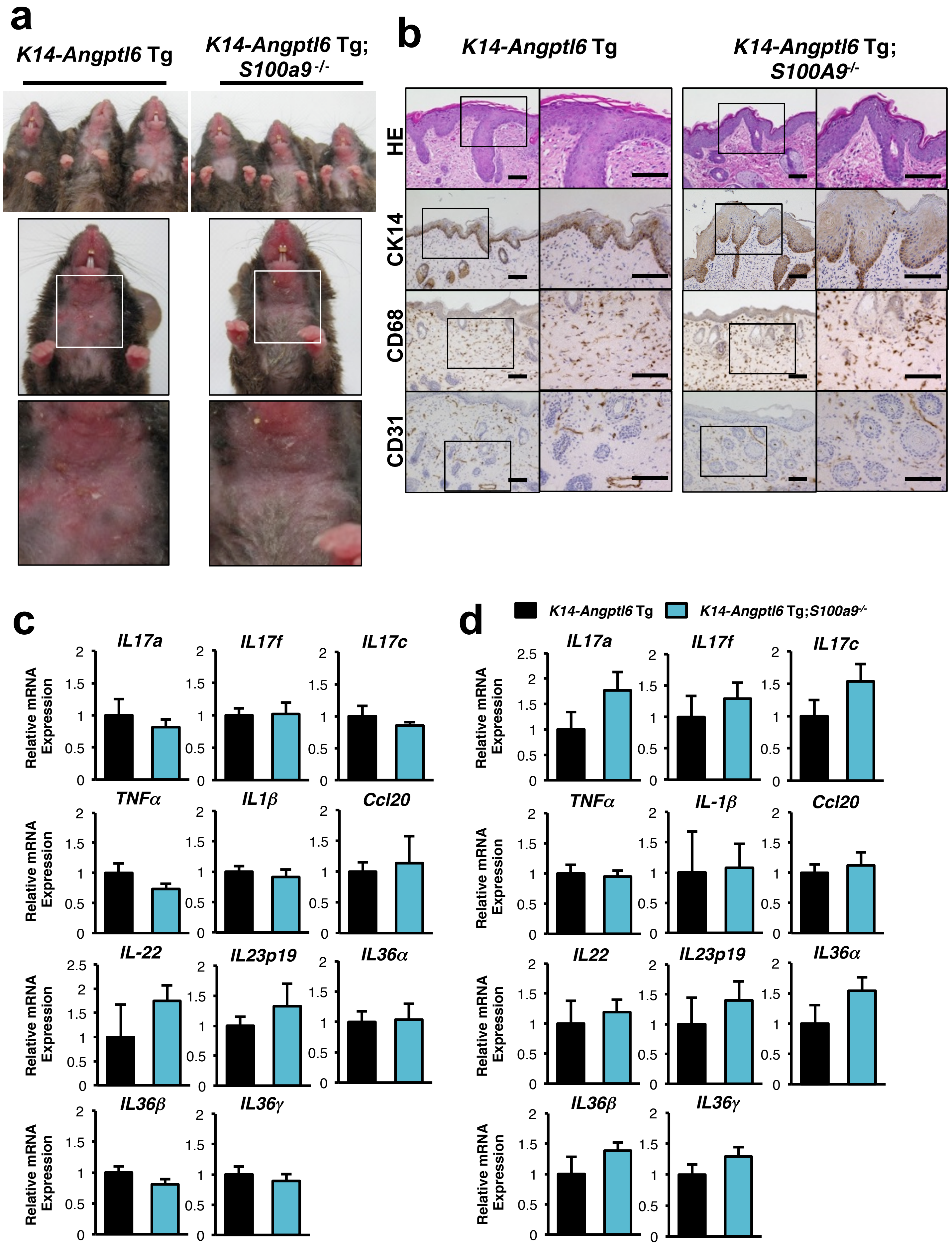

Supplementary Figure S4. Tanigawa *et al.*

Supplementary Figure S4: *K14-Angptl6* Tg;*S100a9*$^{-/-}$ and *K14-Angptl6* Tg female mice show comparable phenotypes

(**a**) Image showing neck skin of *K14-Angptl6* Tg and *K14-Angptl6* Tg;*S100a9*$^{-/-}$ 20-week-old female mice (upper). Magnifications of corresponding regions within white frames are shown in bottom panels. (**b**) HE staining and CK14, CD68 and CD31 staining of neck skin from *K14-Angptl6* Tg (left) and *K14-Angptl6* Tg;*S100a9*$^{-/-}$ (right) female mice in left side. Magnified images of corresponding black-framed regions are shown in right side. Scale bar: 100μm. (**c** and **d**) Quantitative real-time RT-PCR analyses of indicated transcripts in ear (**c**) and neck skin (**d**) samples. Values for respective *K14-Angptl6* Tg female mice were set to 1 (n = 5-7 per each group). Data are means ± SEM.

Supplementary Figure S4. Tanigawa *et al.*

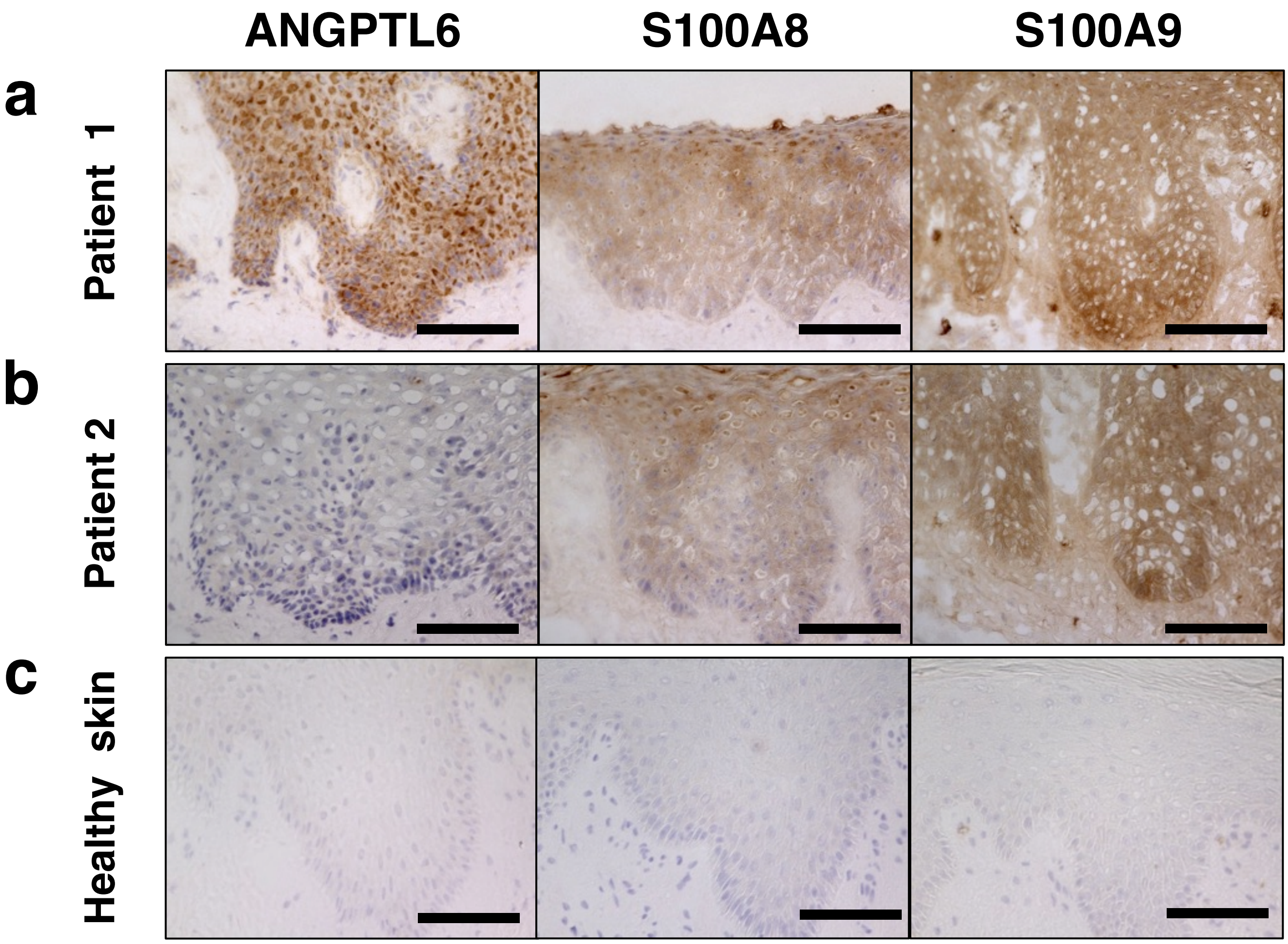

Supplementary Figure S5: ANGPTL6 is expressed in tissue specimens from psoriatic patients

(**a**-**c**) Immunohistochemical staining of serial sections of human skin tissues with antibodies against ANGPTL6, S100A8 and S100A9. Shown are samples from two representative psoriasis patients, including (**a**) Patient 1 (37-year-old female (Case No. 2 in Table 3)) and (**b**) Patient 2 (37-year-old female (Case No.14 in Table 3)). (**c**) Also shown is a sample from a healthy control subject (34-year-old female (Case No. 2 in Table 4)). Each image was magnified from images within black-dotted boxes see in Fig. 4. Scale bar: 100μm.

Supplementary Figure S5. Tanigawa *et al.*
